# The Adolescent Resilience Questionnaire: Validation of a Shortened Version in U.S. Youths

**DOI:** 10.3389/fpsyg.2020.606373

**Published:** 2020-11-27

**Authors:** Jacqueline R. Anderson, Michael Killian, Jennifer L. Hughes, A. John Rush, Madhukar H. Trivedi

**Affiliations:** ^1^Department of Psychiatry, University of Texas Southwestern Medical Center, Dallas, TX, United States; ^2^College of Social Work, Florida State University, Tallahassee, FL, United States; ^3^Duke National University of Singapore, Singapore, Singapore; ^4^Department of Psychiatry, Duke Medical School, Durham, NC, United States; ^5^Department of Psychiatry, Texas Tech Health Science Center, Midland, TX, United States

**Keywords:** adolescence, resilience (psychological), psychometrics, assessment, youth

## Abstract

**Introduction:**

Resilience is a factor in how youth respond to adversity. The 88-item Adolescent Resilience Questionnaire is a comprehensive, multi-dimensional self-report measure of resilience developed with Australian youth.

**Methods:**

Using a cross-sectional adolescent population (*n* = 3,222), confirmatory factor analysis was conducted to replicate the original factor structure. Over half of the adolescents were non-white and 9th graders with a mean age of 15.5.

**Results:**

Our exploratory factor analysis shortened the measure for which we conducted the psychometric analyses. The original factor structure was not replicated. The exploratory factor analysis provided a 49-item measure. Internal consistency reliability for all 12 factors ranged from acceptable (α> 0.70–0.80). The revised factor total scores were highly and significantly correlated with item–total correlation coefficients (*r* > 0.63, *p* < 0.001).

**Conclusion:**

This revised shorter 49-item version of the Adolescent Resilience Questionnaire could be deployed and has acceptable psychometric properties.

## Introduction

Resilience, the ability to adapt to high levels of risk or adversity and achieve positive outcomes (Gartland et al., 2011), is associated with numerous positive psychological, functional, and behavioral outcomes, particularly in youth (Haskett et al., 2006; Farrell et al., 2011). Resilience is a protective factor, reducing the risk of developing mental health disorders such as depression or anxiety (Davydov et al., 2010; Collin-Vézina et al., 2011). Specifically, Sanders et al. (2015) found that greater resilience was associated with better well-being outcomes (e.g., pro-social behaviors, social participation, positive peer group, and educational involvement) in an at-risk youth sample. Further, youth resiliency is associated with fewer internalizing and externalizing symptoms (Hjemdal et al., 2011; Skrove et al., 2013).

Despite the strong relationship between resilience and positive psychological outcomes in adolescents, there is no consensus on how to best assess an adolescent’s resilience (Ahern et al., 2006; Windle et al., 2011; Smith-Osborne and Bolton, 2013). A recent review of self-report resilience measures identified just six out of 68 resilience measures that were appropriate for English-speaking, school-aged youth, with normative data from a United States sample (Vannest et al., 2019). Overall, it was determined there is no consensus as to a “gold standard” measure of resilience (Vannest et al., 2019), several authors have urged for further research into resilience in youth (Ahern et al., 2006; Windle et al., 2011; Smith-Osborne and Bolton, 2013; Vannest et al., 2019).

This report is based on data gathered in a large mental health promotion evaluation study conducted in the United States in which youth resilience was evaluated with the Adolescent Resilience Questionnaire (ARQ). The ARQ was selected for this study because it assesses resilience in multiple domains: individual, family, peers, school, and community.

The ARQ was originally developed as a comprehensive measure of resilience and validated on a sample of Australian adolescents, with the goal of ecological-translation to integrate individual and environment factors that contribute to overall resilience (Gartland et al., 2011). The ARQ was developed through literature reviews, focus groups, and multiple iterations of psychometric testing. The ARQ is comprised of 88 self-report items on a 5-point Likert scale, designed for adolescents between the ages of 11 and 19. The scale has since been translated into multiple languages (e.g., Romanian, Spanish, Persian, and Nepali). Across several studies investigating the psychometrics properties of the ARQ, researchers found mixed results in replicating the origin factor structure. However, when factor structure was replicated or modified (removing subscales), results indicated adequate internal consistency (range from 0.60 to 0.89) (Guilera et al., 2015; Marici, 2015; Cheraghi et al., 2017; Singh et al., 2019).

Based on these mixed results and the dearth of data on US youth, this report aimed to evaluate the psychometric properties of the 88-item ARQ using data obtained from a sample of opportunity. This evaluation suggested that a reduced number of items might form a more stable factor structure. The resulting 49-item ARQ was examined psychometrically through an exploratory factor analysis. This ARQ was also assessed for convergent and discriminant validity, as evidenced by correlations with quality of life and symptoms of psychopathology. Gender differences were explored.

## Materials and Methods

### Study Design and Participants

In the 2017–2018 and 2018–2019 academic years, a mental health promotion program was delivered to 1,000 of high school students in North Texas. This program was an expansion of a two-state study examining the feasibility and acceptability of the mental health promotion program (Lindow et al., 2020). As part of the mental health promotion program, a subset of students (*n* = 3,222) assented to participate in a research study to evaluate the program. A cross-sectional adolescent population completed the research questionnaires during a class period approximately 1 week before beginning the program. The study was approved by the UT Southwestern Institutional Review Board, and parents and students provided written informed consent/assent. From the mental health promotion program study data, the current study examined the students’ ratings of the Adolescent Resilience Questionnaire (ARQ).

### Measures

#### Adolescent Resilience Questionnaire

The ARQ is an 88-item self-report measure for adolescents (11–19 years old) to assess both individual and environmental factors that contribute to resilience (Gartland et al., 2011). The measure assesses resilience from the individual domain and from several environmental domains (family, peers, school, and community). The individual domain measures resilience captured by 5 individual traits: Confidence (8 items), Emotional Insight (8 items), Negative Cognition (8 items), Social Skills (8 items), and Empathy and Tolerance (8 items). The environmental domains of family (11 items), peers (15 items), and school (16 items), are assessed based on the respondent’s engagement in each domain as reflected in the parameters of Connectedness and Availability. The final environmental domain (Community) is assessed by Connectedness (6 items). Each item is responded to on a five-point Likert scale, anchored by *Almost never* (1) and *Almost always* (5). The measure has good internal consistency (range between 0.70 and 0.90) (Gartland et al., 2011). By examining each domain, the ARQ assesses individual traits that contribute to resilience and the level of engagement in different environmental resources that may contribute to resilience in times of adversity. Higher scores indicate greater resilience whereas lower scores would indicate vulnerability to adverse events. Gender differences are similar to other literature, such that boys reported more self-confidence and were less likely to experience negative cognitions (Guilera et al., 2015).

*Pediatric Quality of Life Enjoyment and Satisfaction* (PQ-LES-Q) is a 15-item measure to assess quality of life and life satisfaction in children and adolescents (Endicott et al., 2006). Each item can be rated from 1 (very poor) to 5 (very good). The measure has been found to have good internal consistency of 0.87 (Endicott et al., 2006). Higher scores (scores range 1–65) show greater quality of life and life satisfaction. This measure was used to examine concurrent validity to show those with higher levels of resilience would have greater quality of life.

*Quick Inventory of Depressive Symptomatology–Adolescents* (QIDS-A) is comprised of 17 items, assessing symptoms associated with depression (Bernstein et al., 2010). Internal consistency is high for the self-report version (Cronbach’s alpha = 0.86) (Bernstein et al., 2010). Ranges for interpreting the QIDS-A are as follows: 6–10 for mild depression, 11–15 for moderate, 16–20 for severe, and greater than 21 for very severe depression. This measure was used to demonstrate discriminant validity, to demonstrate that higher resilience scores would correspond to lower levels of depression.

*Generalized Anxiety Disorder 7-item* (GAD-7) is a self-report measure to assess anxiety symptoms (Spitzer et al., 2006). Items responses are rated on a 4-point Likert scale from not at all (0) to nearly every day (3). Internal consistency is high (Cronbach’s alpha = 0.92). Scores of 5–9 indicate mild anxiety, 10–14 indicate moderate anxiety, and 15 or greater indicate severe anxiety. This measure was used to establish discriminant validity by evaluating whether persons with lower resilience scores would have higher levels of anxiety.

### Statistical Analysis

Confirmatory factor analysis were conducted to examine the fit of the ARQ measurement model to the data and adolescents’ responses to items. Using Mplus 8.3 (Muthén and Muthén, 1998–2018), the factor model was tested using a number of indices of model fit. The model chi-square (χ^2^) tests the model variance-covariance matrix against that obtained from the data (the common fit function in SEM, Σ = ΣΘ) with smaller and non-significant χ^2^-values indicating better model fit (Kline, 2016). The Root Mean Square Error of Approximation (RMSEA; Kline, 2016), Comparative Fit Index (CFI; Hu and Bentler, 1999), and Tucker-Lewis Index (TLI; Tucker and Lewis, 1973) were used. CFI and TLI values each greater than 0.95, and RMSEA values lower than 0.08 indicate good model fit (Brown, 2014). The standardized root mean residual (SRMR; Brown, 2014) measures the standardized differences between the model and data variance-covariance matrices, values greater than 0.10 generally indicate poor model fit (Kline, 2016). Cronbach alpha coefficients were obtained to assess the internal consistency reliability of each factor. With model fit being poor with nearly all the original ARQ factors, exploratory factor analyses were undertaken to examine items for lack of fit to each factor. Items were examined and selected for possible removal from each model using theoretical and statistical methods. Authors reviewed each subscale and examined items for face validity with the overarching constructs. Items that did not seem to align with the face validity were flagged to examine statistically. Statistically if item significantly loaded onto multiple factors (i.e., substantially cross-loaded across extracted factors, loading > 0.40), if the item had comparatively high residual variance (i.e., residual variance = 1−*r*^2^, or variance in the item unexplained in the model), and if the removal of the item generated improved model fit based on numerous fit indices.

Various other statistical tests were used to assess the measurement validity of the ARQ including multivariate analysis of variance (MANOVA) to assess differences in ARQ factor scores across genders. Additionally, Pearson correlation coefficients were calculated to examine the association between ARQ factor scores and other self-report measure scores (e.g., symptoms of psychopathology and quality of life) reported by the adolescents.

### Hypotheses

We hypothesized that the 88-item ARQ factor structure would be replicated and have adequate psychometric properties. We hypothesized we could reduce the 88-item ARQ to a shortened version with adequate psychometric properties.

## Results

### Demographic Characteristics

Table 1 summarizes the socio-demographic characteristics of the sample. Over half were non-white and 9th graders. Over two-thirds were in a two parent home. The average age of the sample was 15.5 years.

**TABLE 1:** Demographics.

**Variables**	**%**
**Grade**	
*7th to 8th grade*	2.5%
*9th grade*	47.9%
*10th grade*	27.8%
*11th grade*	15.4%
*12th grade*	6.4%
**Gender**	
*Female*	61.5%
**Lives with**	
*I always live with my mother and father*	69.4%
*I live only with my mother*	14.3%
*I live only with my father*	2.2%
*Other*	14.1%
US citizen, yes	93.9%
English native/primary language, yes	71.0%
**Race**	
*Black or African American*	10.8%
*Asian*	15.3%
*White*	42.7%
*More than one race*	10%
*Other*	21.2%
Hispanic, Latino, or Spanish origin, yes	31.8%

### ARQ Validity and Reliability

#### Confirmatory Factor Analysis (CFA)

Responses from 3,222 adolescents on the ARQ were used in CFA modeling examining the factor validity of each factor from the Individual, Family, Peer, School, and Community domains (see Table 2). Overall, the ARQ measurement model poorly fitted the data, except for the Negative Cognition factor within the Individual domain (χ^2^ = 289.66, *df* = 20, *p* < 0.001; RMSEA = 0.065, 95%; CI = 0.058, 0.071; CFI = 0.990, TLI = 0.986; SRMR = 0.018; standardized loadings range 0.582–0.824, α = 0.874). Alternative models were examined including all factors from each domain in correlated factor models (e.g., including all five factors from the Individual domain as correlated latent variables). Results again indicated poor fit of the correlated factor models for the factors within each the Individual, Family, Peer, and School domains (Table 2).

**TABLE 2:** Original and revised ARQ factor and measurement models through exploratory factor analysis (*n* = 3,222).

**Model**	**Model version**	**χ^2^(df)**	**RMSEA**	**90%CI RMSEA**	**CFI**	**TLI**	**SRMR**	**STD loadings**	**α**
**Individual**									
Confidence	Original	1174.264*** (20)	0.134	0.127, 140	0.969	0.957	0.032	0.510–831	0.886
	New 5-item	54.086*** (5)	0.055	0.042, 0.069	0.997	0.994	0.011	0.538–0.885	0.825
Emotional insight	Original	1221.470*** (20)	0.137	0.130, 143	0.886	0.841	0.048	0.456–0.694	0.774
	New 4-item	20.730*** (2)	0.054	0.035, 0.076	0.996	0.989	0.009	0.525–0.780	0.719
Negative cognition	Original	289.659*** (20)	0.065	0.058, 0.071	0.990	0.986	0.018	0.582–0.824	0.874
	New 5-item	69.071*** (5)	0.063	0.050, 0.077	0.996	0.992	0.010	0.704–0.830	0.846
Social skills	Original	963.602*** (20)	0.121	0.115, 0.128	0.942	0.919	0.042	0.414–0.826	0.806
	New 4-item	0.993 (2) [NS]	0.000	0.000, 0.029	10.000	10.000	0.004	0.635–0.744	0.762
Empathy/tolerance	Original	1107.037*** (20)	0.130	0.123, 0.136	0.800	0.721	0.059	0.272–0.706	0.630
	New 4-item	47.177*** (2)	0.084	0.064, 0.105	0.984	0.953	0.017	0.407–0.870	0.614
**Family**									
Connectedness	Original	1932.404*** (20)	0.174	0.168, 0.181	0.968	0.955	0.040	0.358–0.887	0.903
	New 4-item	4.372 (2) [NS]	0.019	0.000, 0.045	10.000	10.000	0.002	0.785–0.902	0.888
Availability	Original	–	–	–	–	–	–	0.737–0.976	0.873
**Peers**									
Connectedness	Original	797.267*** (14)	0.133	0.125, 0.141	0.955	0.933	0.037	0.638–0.843	0.835
	New 4-item	4.372 (2) [NS]	0.019	0.000, 0.045	10.000	10.000	0.002	0.785–0.902	0.888
Availability	Original	1610.281*** (20)	0.159	0.152, 0.165	0.919	0.887	0.059	0.389–0.893	0.805
	New 4-item	14.281*** (2)	0.044	0.025, 0.067	0.997	0.992	0.016	0.618–0.747	0.716
**School domain**									
Supportive environment	Original	868.203*** (14)	0.139	0.132, 0.147	0.945	0.917	0.039	0.568–0.807	0.822
	New 4-item	2.415 (2) [NS]	0.008	0.000, 0.037	10.000	10.000	0.006	0.611–0.826	0.741
Connectedness	Original	3937.156*** (20)	0.250	0.244, 0.257	0.853	0.794	0.097	0.240–0.865	0.772
	New 4-item	31.081*** (2)	0.068	0.048, 0.065	0.999	0.996	0.001	0.294–0.921	0.757
	3-item option	–	–	–	–	–	–	0.708–0.917	0.842
**Community domain**									
Connectedness	Original	979.653*** (9)	0.186	0.177, 0.196	0.975	0.959	0.037	0.756–0.908	0.891
	New 4-item	30.439*** (2)	0.068	0.048, 0.090	0.999	0.997	0.0065	0.770–0.933	0.864

***p < 0.001.

#### Exploratory Factor Analysis (EFA)

Given the poor fit of the data to the originally validated model containing 88 items, exploratory analyses were conducted to examine the measurement model of the ARQ. In order to improve model fit to the data, decrease respondent burden, and increase clinical utility, EFA was conducted on each factor to create a shortened version comprised of 49 items of the ARQ (ARQ_49_). Items were selected for removal from the model if they substantially cross-loaded across extracted factors (loading > 0.40), if the item had comparatively high residual variance (i.e., residual variance = 1−*r*^2^), and if the removal of the item generated improved model fit based on numerous fit indices.

Table 2 shows the EFA results and the revised factor models. In each case, dropping several items created a more concise measure of each factor within each domain. Through the removal of three or four items, model fit substantially improved and demonstrated excellent fit of the data to the revised measurement models within the ARQ. Internal consistency reliability for all factors ranged from acceptable (α > 0.70) to excellent (α > 0.80) except for Individual Empathy/Tolerance (α = 0.614). Compared to the original scores for each factor, the revised factor total scores were significantly and very highly correlated with item-total correlation coefficients ranging from *r* = 0.836 to 0.970 (each *p* < 0.001). Overall, the original ARQ total score from the 88-item version remained very highly correlated with the revised, shortened version (*r* = 0.977, *p* < 0.001), demonstrating comparability of scores from the two versions. The ARQ_49_ items are listed in Tables 3, 4.

**TABLE 3:** Adolescent resilience questionnaire short form individual factor items.

**Confidence**	I am confident that I can achieve what I set out to do
(*M* = 3.72, *SD* = 0.78)	I feel confident that I can handle whatever comes my way
	I am a person who can go with the flow
	I feel confident to do things by myself
	If I have a problem I can work it out
**Emotional insight**	When I am feeling down, I take extra special care of myself
(*M* = 3.41, *SD* = 0.84)	I look for what I can learn out of bad things that happen
	If I have a problem, I know there is someone I can talk to
	If I can’t handle something I find help
**Negative cognition**	I just can’t let go of bad feelings
(*M* = 3.22, *SD* = 0.96)	I can’t stop worrying about my problems
	I tend to think the worst is going to happen
	I dwell on the bad things that happen
	My feelings are out of my control
**Social skills**	I find it hard to express myself to others
(*M* = 3.30, *SD* = 0.94)	I can share my personal thoughts with others
	I can express my opinions when I am in a group
	I find it easy talking to people my age
**Empathy**	I am patient with people who can’t do things as well as I can
(*M* = 3.47, *SD* = 0.74)	I get frustrated when people make mistakes
	I am easily frustrated with people
	I expect people to live up to my standards

**TABLE 4:** Adolescent resilience questionnaire short form family, peer, school, and community factor items.

**Family**	**Connectedness**	I do fun things with my family
	(*M* = 3.64, *SD* = 1.01)	We do things together as a family
		My family understands my needs
		I get to spend enough time with my family
	**Availability**	There is someone in my family I can talk to about anything
	(*M* = 3.86, *SD* = 1.18)	If I have a problem there is someone in my family I can talk to
		There is someone in my family that I feel particularly close to
**Peers**	**Connectedness**	When I am down I have friends that help cheer me up
	(*M* = 4.08, *SD* = 0.82)	I have a friend I can trust with my private thoughts and feelings
		I have friends who make me laugh
		I get to spend enough time with my friends
	**Availability**	I feel left out of things
	(*M* = 3.60, *SD* = 0.85)	I wish I had more friends I felt close to
		I find it hard to stay friends with people
		I am happy with my friendship group
**School**	**Supportive environment**	My teachers are caring and supportive of me
	(*M* = 3.48, *SD* = 0.91)	My teachers provide me with extra help if I need it
		My teachers notice when I am doing a good job and let me know
		There is an adult at school who I could talk to if I had a personal problem
	**Connectedness**	I hate going to school
	(*M* = 2.94, *SD* = 0.98)	I am bored at school
		My teachers expect too much of me
		I enjoy going to school
**Community**	**Connectedness**	People in my neighborhood are caring
	(*M* = 3.34, *SD* = 1.06)	The people in my neighborhood treat other people fairly
		I like my neighborhood
		The people in my neighborhood look out for me

### ARQ_49_ Scores and Correlates

Individual subscale correlations were conducted (Table 5). Individual subscales were correlated to quality of life and symptoms of psychopathology. Peer, Family, School, and Community subscales were investigated. These subscales were also correlated to quality of life and symptoms of psychopathology.

**TABLE 5:** Adolescent resilience questionnaire factor scores and correlates.

	**Anxiety**	**Quality of life**	**Depression**
**Individual**			
Confidence	−0.485***	0.623***	−0.549***
Emotional insight	−0.433***	0.633***	−0.502***
Negative cognition	−0.676***	0.603***	−0.646***
Social skills	−0.424***	0.563***	−0.490***
Empathy/tolerance	−0.269***	0.262***	−0.268***
**Family**			
Connectedness	−0.433***	0.658***	−0.518***
Availability	−0.289***	0.491***	−0.371***
Peers			
Connectedness	−0.245***	0.481***	−0.330***
Availability	−0.393***	0.522***	−0.458***
**School domain**			
Supportive environment	−0.272***	0.521***	−0.371***
Connectedness	−0.333***	0.477***	−0.403***
Community domain			
Connectedness	−0.269***	0.458***	−0.359***

***p < 0.001.

#### Gender

Minimal gender differences were found. ARQ_49_ scores were used in a MANOVA model with gender as a predictor. ARQ_49_ scores significantly differed among female and male adolescents [Wilk’s Lambda = 0.905, *F*(12, 3,012) = 26.34, *p* < 0.001, partial η^2^ = 0.095], though the effect size of gender across all ARQ scores was small. Specifically, male adolescents reported greater Individual Confidence [*F*(1, 3,023) = 91.242, *p* < 0.001, η^2^ = 0.029], Emotional Insight [*F*(1, 3,023) = 13.672, *p* < 0.001, η^2^ = 0.005], Negative Cognition [*F*(1, 3,023) = 138.039, *p* < 0.001, η^2^ = 0.044], Social Skills [*F*(1, 3,023) = 43.118, *p* < 0.001, η^2^ = 0.014], Peer Availability [*F*(1, 3,023) = 29.252, *p* <0.001, η^2^ = 0.01], and School Connectedness [*F*(1, 3,023) = 5.159, *p* = 0.023, η^2^ = 0.002]. Female adolescents reported greater Peer Connectedness [*F*(1, 3,023) = 16.9, *p* < 0.001, η^2^ = 0.006] and Family Connectedness [*F*(1, 3,023) = 11.758, *p* = 0.001, η^2^ = 0.004]. No significant differences were reported on Community Connectedness [*F*(1, 3,023) = 2.014, *p* = 0.156, η^2^ = 0.001], School Support Environment [*F*(1, 3,023) = 1.044, *p* = 0.307, η^2^ = 0], and Family Availability [*F*(1, 3,023) = 0.085, *p* = 0.771, η^2^ = 0], and Individual Empathy/Tolerance [*F*(1, 3,023) = 0.001, *p* < 0.975, η^2^ = 0]. All effect sizes were small (η^2^ = 0.001–0.044) for gender differences.

#### Pediatric Quality of Life Enjoyment and Satisfaction (PQ-LES-Q)

Quality of life and satisfaction scores from the PQ-LES-Q measure were significantly and strongly correlated with ARQ_49_ Individual subscale (Confidence, Emotional Insight, Negative Cognition, Social Skills) scores (*r* = 0.563–0.633, each *p* < 0.001) except for Individual Empathy/Tolerance which was weakly correlated (*r* = 0.262, *p* < 0.001). ARQ_49_ Family, Peer, School, and Community factor scores were similarly strongly correlated with PQ-LES-Q scores (*r* = 0.458–0.658, each *p* < 0.001). Higher ARQ_49_ scores were associated with adolescent reports of greater quality of life and satisfaction.

#### Depression and Anxiety

QIDS-A depressive symptom severity scores were significantly and negatively correlated with ARQ_49_ scores within the Individual subscales (*r* = −0.268 to −0.646, each *p* < 0.001), Family (*r* = −0.371 to −0.518, each *p* < 0.001), Peer (*r* = −0.330 to −0.458, each *p* <0.001), School (*r* = −0.371 to −0.403, each *p* <0.001), and Community factor scores (*r* = −0.359, *p* < 0.001). Similarly, GAD-7 scores were significantly and negatively correlated Individual (*r* = −0.269 to −0.676, each *p* < 0.001), Family (*r* = −0.289 to −0.433, each *p* < 0.001), Peer (*r* = −0.245 to −0.393, each *p* < 0.001), School (*r* = −0.272 to −0.333, each *p* < 0.001), and Community factor scores (*r* = −0.269, *p* < 0.001). Lower ARQ_49_ scores were associated with greater reports of depression and anxiety.

## Discussion

This study evaluated the ARQ on a novel United States youth population (Gartland et al., 2011). Our initial confirmatory factor analysis did not support the factorial structure implied by the 12 scales structure provided by the developers (Gartland et al., 2011). Other studies have also had difficulty replicating the exact factor structure from the original study (Guilera et al., 2015; Marici, 2015; Cheraghi et al., 2017; Singh et al., 2019). Due to the difficulties of replicating the factor structure, we conducted exploratory analyses, which resulted in the ARQ_49_ with good fit and adequate internal consistency.

The ARQ_49_ had high correlations with the original 88-item version of the ARQ. After the measure revisions, gender differences were explored and small differences were found similar to the previous studies (Guilera et al., 2015; Marici, 2015; Cheraghi et al., 2017; Singh et al., 2019). For each subscale, internal consistencies were adequate to good. Subscales were examined for convergent (quality of life) and discriminant validity (symptoms of psychopathology), and they were determined to be adequate.

This is the first study to our knowledge to explore the psychometric properties of the ARQ in a United States population, and to develop and validate a briefer version of the measure, the ARQ_49_. The ARQ_49_ also reduces respondent burden. The ARQ_49_ psychometrics meet the strong standards recommended by several meta-analyses (Ahern et al., 2006; Windle et al., 2011; Smith-Osborne and Bolton, 2013; Vannest et al., 2019). Therefore, ARQ_49_ is a seventh measure of resilience validated on the United States youth population with adequate psychometric properties. One of the strengths of the scale is that it is a very comprehensive measure that examines not only individual traits, but also environmental factors that are likely to affect resilience. In addition to developing a shortened version of this measure, another strength of this study is the large sample size of diverse students from different types of schools across North Texas. Further, the current findings are similar not only to other ARQ studies, but similar to other resilience study’s to indicate resilience as a protective factor.

Clinicians should use this measure when wanting to measure resilience in youth across multiple domains (Family, Peers, School, and Community) to obtain a comprehensive understanding of the youth’s overall resilience. Further, clinicians could examine the different domains of resilience within the external or internal factors as targets of intervention or resilience development. For example, if a clinician noticed a student’s scores are normal in all areas except school connectedness, then the clinician could look for interventions to promote school connectedness with that student. Alternatively, a clinician could use a strength-based approach to highlight the higher scored domains with a student to create a change plan by using the student’s strengths. Beyond clinician usage, a school system could use this measure to assess their student body’s overall and domain specific resilience alongside a universal prevention or social emotional curriculum to understand how the curriculum promotes mental health. Additionally, resilience is important to understand short and long-term outcomes, the shortening of this measure may promote the use of this measure by decreasing the amount of time to complete it (Davydov et al., 2010; Collin-Vézina et al., 2011; Turner et al., 2017). Thus, ARQ_49_ could also be incorporated into assessments to understand mediators of trauma and development of psychopathology.

A limitation of this study is there was not another resilience measure in this assessment to use as a comparison with the ARQ_49__._ Thus, it is unclear whether the ARQ_49_ is directly related to an established resilience measure validated in the United States. Due to the broad definition of resilience in the ARQ with external and internal factors, future studies should examine the relationship of the ARQ_49_ with another well-established measures of resilience in the United States such as six identified in the recent review (Vannest et al., 2019). The study included a cross-sectional population of adolescents in North Texas, so it may not be generalizable to the entire North American population. Replication of these findings with other youth residing in the United States is needed to further understand how the resilience measure is relevant in different regions. Finally, resilience is defined as the ability to overcome adversity (Gartland et al., 2011). However, the current study sample was from a general population, so it unclear whether the students sampled have had adverse experiences that would lead to the development of resilience. In future studies, researchers could use the ARQ_49_ to examine how adverse experiences lead to the development of higher or lower resilience scores.

Overall, the ARQ_49_ provides a comprehensive tool that assesses individual traits and environmental aspects of adolescent resilience with strong psychometric properties appropriate for adolescents in the United States.

## Data Availability Statement

The original contributions presented in the study are included in the article/supplementary material, further inquiries can be directed to the corresponding author.

## Ethics Statement

The studies involving human participants were reviewed and approved by the University of Texas Southwestern Medical Center Institutional Review Board. Written informed consent to participate in this study was provided by the participants’ legal guardian, and written informed assent was provided by the participant.

## Author Contributions

JA, JH, and MT conceived and designed the analyses. MK conducted the analyses. JH and MT oversaw the data collection and the related project. JA, AR, and MK wrote the manuscript with contribution from the other authors. All authors contributed to the article and approved the submitted version.

## Conflict of Interest

JH has served as Youth Aware of Mental Health (YAM) trainer, consulting for Mental Health in Mind International. JH also receives royalties from Guilford Press. AR has received consulting fees from Compass Inc., Curbstone Consultant LLC, Emmes Corp., Evecxia Therapeutics, Inc., Holmusk, Johnson and Johnson (Janssen), Liva-Nova, Neurocrine Biosciences Inc., Otsuka-US, Sunovion; speaking fees from Liva-Nova, Johnson and Johnson (Janssen); and royalties from Guilford Press and the University of Texas Southwestern Medical Center, Dallas, TX (for the Inventory of Depressive Symptoms and its derivatives). He is also named co-inventor on two patents: U.S. Patent No. 7,795,033: Methods to Predict the Outcome of Treatment with Antidepressant Medication, Inventors: McMahon FJ, Laje G, Manji H, Rush AJ, Paddock S, Wilson AS; and U.S. Patent No. 7,906,283: Methods to Identify Patients at Risk of Developing Adverse Events During Treatment with Antidepressant Medication, Inventors: McMahon FJ, Laje G, Manji H, Rush AJ, Paddock S. MT has served as an adviser or consultant for ACADIA Pharmaceuticals, Alkermes Inc, Allergan, Alto Neuroscience Inc., Applied Clinical Intelligence, LLC, Axsome Therapeutics, Boegringer Ingelheim, GreenLight VitalSign6 Inc., Janssen Pharmaceutical, Jazz Pharmaceutical, Lundbeck Research USA, Medscape, Merck Sharp & Dohme Corp., Myriad Neuroscience, Navitor, Otsuka America Pharmaceutical Inc., Perception Neuroscience Holdings, Pharmerit International, SAGE Therapeutics, Signant Health, Takeda Pharmaceuticals Inc.; he has received grants or research support from the NIMH, NIDA, the Cancer Prevention and Research Institute of Texas, Patient-Centered Outcomes Research Institute, and Janssen; and has received editorial compensation from Healthcare Global Village and Engage Health Media. The remaining authors declare that the research was conducted in the absence of any commercial or financial relationships that could be construed as a potential conflict of interest.
